# Epidermal growth factor receptor inhibitor with fluorouracil, leucovorin, and irinotecan as an alternative treatment for advanced upper tract urothelial carcinoma: a case report

**DOI:** 10.1186/s13256-016-0879-6

**Published:** 2016-04-18

**Authors:** Yen-Man Lu, Tsu-Ming Chien, Chih-Hung Lin, Chee-Yin Chai, Chun-Nung Huang

**Affiliations:** 1 Department of Urology, Kaohsiung Medical University Hospital, No. 100, Tz-You 1st Road, Kaohsiung, 807 Taiwan; 2 Department of Urology, School of Medicine, College of Medicine, Kaohsiung Medical University, Kaohsiung, Taiwan; 3 Department of Pathology, Kaohsiung Medical University Hospital, Kaohsiung Medical University, Kaohsiung, Taiwan; 4 Graduate Institute of Medicine, College of Medicine, Kaohsiung Medical University, Kaohsiung, Taiwan; 5 Department of Pathology, Faculty of Medicine, College of Medicine, Kaohsiung Medical University, Kaohsiung, Taiwan; 6 Institute of Biomedical Sciences, National Sun Yat-Sen University, Kaohsiung, Taiwan

**Keywords:** Urothelial carcinoma, Upper tract urothelial carcinoma, Chemotherapy, Epidermal growth factor receptor, Target therapy, Surgery

## Abstract

**Background:**

Currently, there is no standard salvage regimen after the failure of cisplatin-based chemotherapy for advanced urothelial carcinoma. The combination of epidermal growth factor receptor inhibitor with fluorouracil, leucovorin, and irinotecan was originally designed for the treatment of metastatic colorectal cancer. Until now, there have been no reports using this combination therapy in treating advanced upper tract urothelial carcinoma. To the best of our knowledge, this is the first report showing this possible treatment regimen for advanced upper tract urothelial carcinoma.

**Case presentation:**

We report the case of a 90-year-old Chinese woman who was diagnosed with metastatic colorectal cancer and urothelial carcinoma of the bladder, ureter, and renal pelvis. The upper tract urothelial carcinoma was well controlled by the chemotherapy regimen for metastatic colorectal cancer. Considering her age, we used only laser ablation for the treatment of her urothelial carcinoma in combination with intravesical mitomycin C chemotherapy. Follow-up cystoscopy and ureterorenoscopy showed an unexpected regression of the upper tract urothelial tumor. Contrast-enhanced computed tomography also demonstrated the same results.

**Conclusions:**

This novel regimen for the treatment of upper tract urothelial carcinoma may merit further investigation or evaluation in clinical trials.

**Electronic supplementary material:**

The online version of this article (doi:10.1186/s13256-016-0879-6) contains supplementary material, which is available to authorized users.

## Background

Upper tract urothelial carcinomas (UTUCs) are tumors derived from the urothelium along the pyelocaliceal cavities and ureters, and they account for only 5–10 % of urinary tract carcinomas [1]. Radical nephroureterectomy (RNU) with bladder cuff excision, using either open or laparoscopic methods, is the gold standard treatment for adequate local tumor control and better long-term survival [2]. Despite optimal surgical treatment, patient outcomes are still not satisfactory. Until now, no definitive evidence has existed regarding the use of perioperative chemotherapy in the management of UTUC. The current practice guideline is almost entirely derived from research related to muscle-invasive bladder cancer [3]. Though cisplatin-based chemotherapy is the current first-line therapy regimen, its efficacy remains poorly defined [3]. Furthermore, there is no standard salvage regimen after the failure of cisplatin-based chemotherapy. We recently treated a double cancer case with metastatic colorectal cancer and urothelial carcinoma of the bladder, ureter, and renal pelvis. The UTUC was well controlled by the chemotherapy regimen for metastatic colorectal cancer.

## Case presentation

A 90-year-old Chinese woman with a complaint of bloody stool was admitted to our department of general surgery to undergo surgery for colorectal cancer. She also mentioned intermittent painless gross hematuria with blood clots for months. She has been taking hypertension medication (angiotensin-converting-enzyme inhibitor) for more than 20 years. She had never smoked cigarettes and denied chemical exposure, herb use, or groundwater use. Contrast-enhanced abdominal computed tomography (CT) showed sigmoid colon cancer with peritoneal carcinomatosis and para-aortic lymph node metastasis. Imaging of her urinary tract revealed bilateral renal pelvis, bilateral ureter, and bladder urothelial carcinoma.

Because of our patient’s advanced age and acceptable renal function, aggressive surgical treatment was not a first priority for the treatment of her urothelial carcinoma. After discussing the treatment plan with our patient and her family, we performed a urinary tract tumor biopsy and laser ablation. We then performed a robotic-assisted sigmoid colectomy using the da Vinci (Intuitive Surgical, Mountain View, CA, USA) system, and the pathologic report indicated a diagnosis of pT3N2M0. Examination of her urinary tract revealed a high-grade infiltrating urothelial carcinoma of the bladder and ureter. No complications were observed during the operation. Under a generally stable condition, our patient was discharged on the fourth day after her operation.

**Fig. 1:**
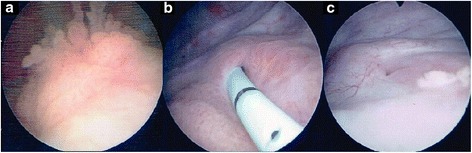
Cystoscopy (**a**–**c**) shows that the bladder tumor had shrunk. The three pictures were taken over the course of the treatment

**Fig. 2:**
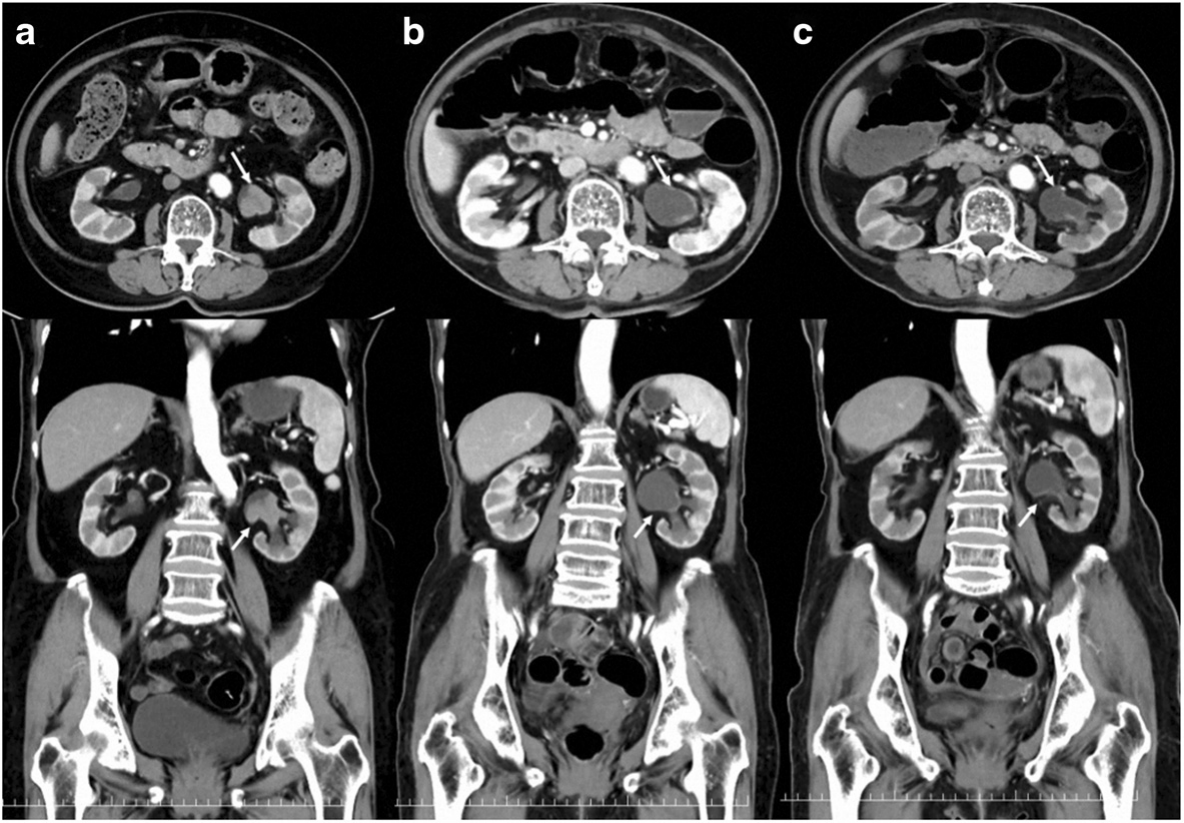
Repeated contrast-enhanced computed tomography (**a**–**c**) shows that the left pelvic tumor, which showed no mutation (wild type), had shrunk (*white arrow*). The three pictures were taken over the course of the treatment

Analysis of codons 12 and 13 of the *KRAS* gene in the excised colorectal cancer tissues revealed that they were mutation-negative (wild type) (Additional file 1 to 3 : Figure S1 to S3 and Additional file 4). Therefore, nine cycles of adjuvant chemotherapy was initiated with cetuximab (Erbitux; Bristol-Myers Squibb, Princeton, NJ, USA) and fluorouracil, leucovorin, and irinotecan (FOLFIRI) for the treatment of the metastatic colorectal cancer. As for the urothelial carcinoma of the bladder, intravesical mitomycin C therapy was administered five times. Our patient tolerated the chemotherapy well. Cystoscopy, ureterorenoscopy, and urine cytology examinations were performed every 3 months. The size of the bladder tumor became smaller (Fig. 1). Surprisingly, a repeat contrast-enhanced abdominal CT showed shrinkage of the renal pelvis tumor (Fig. 2), and her hematuria also improved.

Further analysis of the urothelial carcinoma of the urinary tract was carried out, and the results showed a wild-type *KRAS* gene in the urothelial carcinoma tissues.

As of 20 months after the operation, our patient has a good stable condition without progression of either her colorectal cancer or urothelial cancer.

## Discussion

Currently, there is no standard salvage regimen after the failure of cisplatin-based chemotherapy for advanced urothelial carcinoma. Many novel agents have been developed and have shown modest activity in urothelial carcinoma. With the advancement of molecular biology and a deeper understanding of the pathogenesis of urothelial carcinoma, new approaches for treating patients using molecularly targeted therapies have emerged. Previous studies have shown that the over-expression of epidermal growth factor receptor (EGFR) predicts poor survival and stage progression [4, 5]. EGFR is more strongly expressed in invasive tumors (pT2–T4) and high-grade tumors than in superficial or low-grade tumors [4]. However, mutations within the kinase domain and truncations of the EGFR are rarely seen in bladder cancer, and they have emerged as attractive therapeutic targets. Cetuximab (an EGFR inhibitor) is a chimeric human/mouse monoclonal antibody that prevents dimerization by binding to the extracellular domain of EGFR. Wong *et al*. [6] conducted a phase II clinical trial to evaluate the efficacy of cetuximab with or without paclitaxel as salvage chemotherapy in patients with previously treated advanced urothelial cancer. The study was closed after 8 weeks, because 9 of 11 patients had disease progression in the paclitaxel-alone arm. Progression-free survival greater than 16 weeks was noted in 12 of 28 patients in the combination arm. They concluded that cetuximab may augment the activity of paclitaxel, and the combination merits further study as salvage chemotherapy in patients with previously treated urothelial carcinoma. Although the mechanisms are unclear, it seems that cetuximab is not effective for use as a single-agent therapy. Another phase II clinical trial (clinicaltrials.gov ID: NCT00645593) is investigating this augmentation by evaluating the effect of gemcitabine and cisplatin chemotherapy either in combination with cetuximab or not in patients with locally advanced or metastatic urothelial cancer (above stage T4b), and results are pending. To date, there have been at least eight clinical trials aimed at treating urothelial carcinoma by targeting EGFR (https://clinicaltrials.gov/ct2/results?term=Bladder+cancer+AND+epidermal+growth+factor+receptor&Search=Search).

Despite the widespread implementation of traditional cisplatin-based chemotherapy, other clinical trials have included synchronous chemotherapy using fluorouracil and mitomycin C combined with radiotherapy. These trials demonstrated significant improvements in the control of bladder cancer compared with radiotherapy alone [7]. Gemcitabine-based and carboplatin-based regimens have also been studied; Sio *et al*. [8] demonstrated a 61 % response rate with the combination of gemcitabine and irinotecan.

The Cancer Genome Atlas project performed an integrated analysis of 131 urothelial carcinoma cases to provide a comprehensive landscape of molecular alterations [9]. They determined that 69 % of the tumors could potentially be treated, through the use of therapy targeted to the phosphatidylinositol-3-OH kinase/AKT/mTOR pathway in 42 % of the tumors or targeted to the RTK/MAPK pathway (including ERBB2) in 45 % of the tumors. They also found that urothelial carcinoma had more frequent mutations in chromatin regulatory genes. The current findings all suggest the future possibility of targeted therapy. The development of reliable molecular markers is expected to make personalized treatment strategies possible for the treatment of urothelial carcinoma. In our case, the unexpected treatment effect of cetuximab and FOLFIRI in urothelial carcinoma has not previously been reported. The urothelial carcinoma in this case harbored the wild-type form of *KRAS*, indicating its susceptibility to cetuximab.

## Conclusions

The use of EGFR inhibitors combined with chemotherapy for the treatment of UTUC may merit further investigation or evaluation in clinical trials.

## Consent

Written informed consent was obtained from the patient for publication of this case report and any accompanying images. A copy of the written consent is available for review by the Editor-in-Chief of this journal.

## Additional files

Additional file 1: Figure S1. (A) Photomicrograph of the rectum showed the presence of neoplastic cells resembling adenocarcinoma (arrow). (B) Microscopic features of renal pelvis revealed infiltrating urothelial carcinoma, high grade (arrow). (C) Microscopic features of ureter revealed infiltrating urothelial carcinoma, high grade (arrow). (D) Microscopic features of bladder revealed infiltrating urothelial carcinoma, high grade (arrow). A–D show hematoxylin and eosin stain, magnification ×200. (TIF 2307 kb)

Additional file 2: Figure S2. Electropherogram of the telomerase reverse transcriptase (TERT) promoter region. (TIF 3855 kb)

Additional file 3: Figure S3. Electropherogram of exon 2 of the *KRAS* gene. (TIF 3851 kb)

Additional file 4: Supporting information. (DOCX 16 kb)
