# Supplementary figures and images for: Epidermal growth factor receptor inhibitor with fluorouracil, leucovorin, and irinotecan as an alternative treatment for advanced upper tract urothelial carcinoma: a case report
Source: J Med Case Rep. 2016 Apr 18;10:98. doi: 10.1186/s13256-016-0879-6 (PMC4835853; doi:10.1186/s13256-016-0879-6)

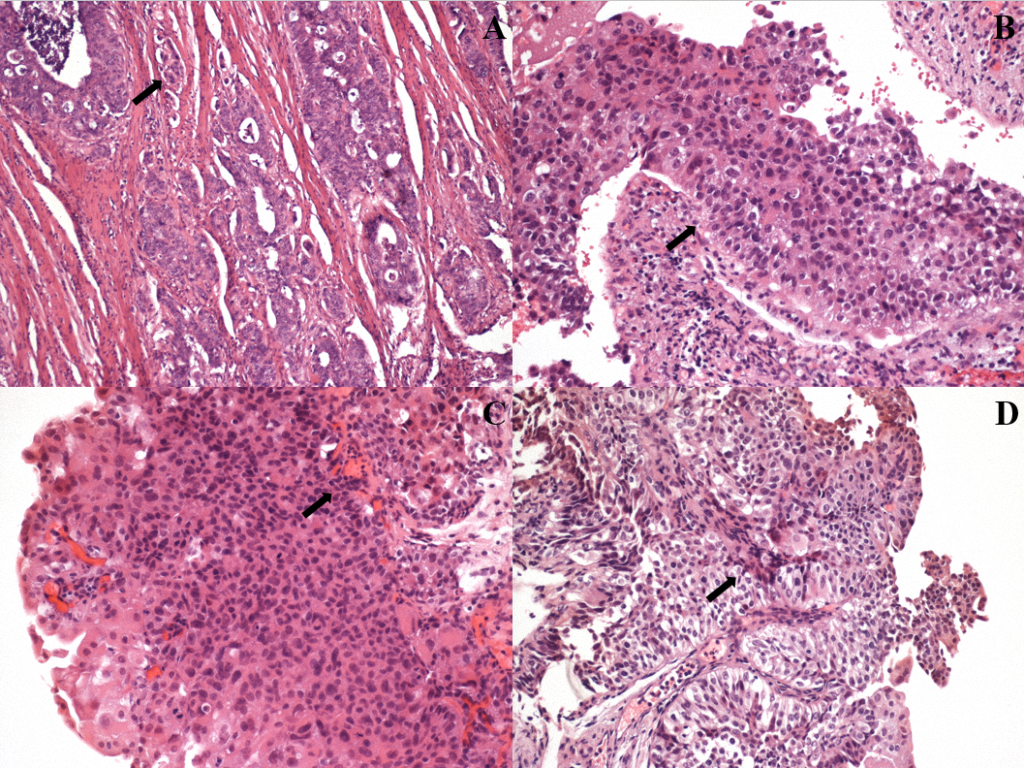

Supplement: Additional file 1: Figure S1. — (A) Photomicrograph of the rectum showed the presence of neoplastic cells resembling adenocarcinoma (arrow). (B) Microscopic features of renal pelvis revealed infiltrating urothelial carcinoma, high grade (arrow). (C) Microscopic features of ureter revealed infiltrating urothelial carcinoma, high grade (arrow). (D) Microscopic features of bladder revealed infiltrating urothelial carcinoma, high grade (arrow). A–D show hematoxylin and eosin stain, magnification ×200. (TIF 2307 kb) [file 13256_2016_879_MOESM1_ESM.tif]

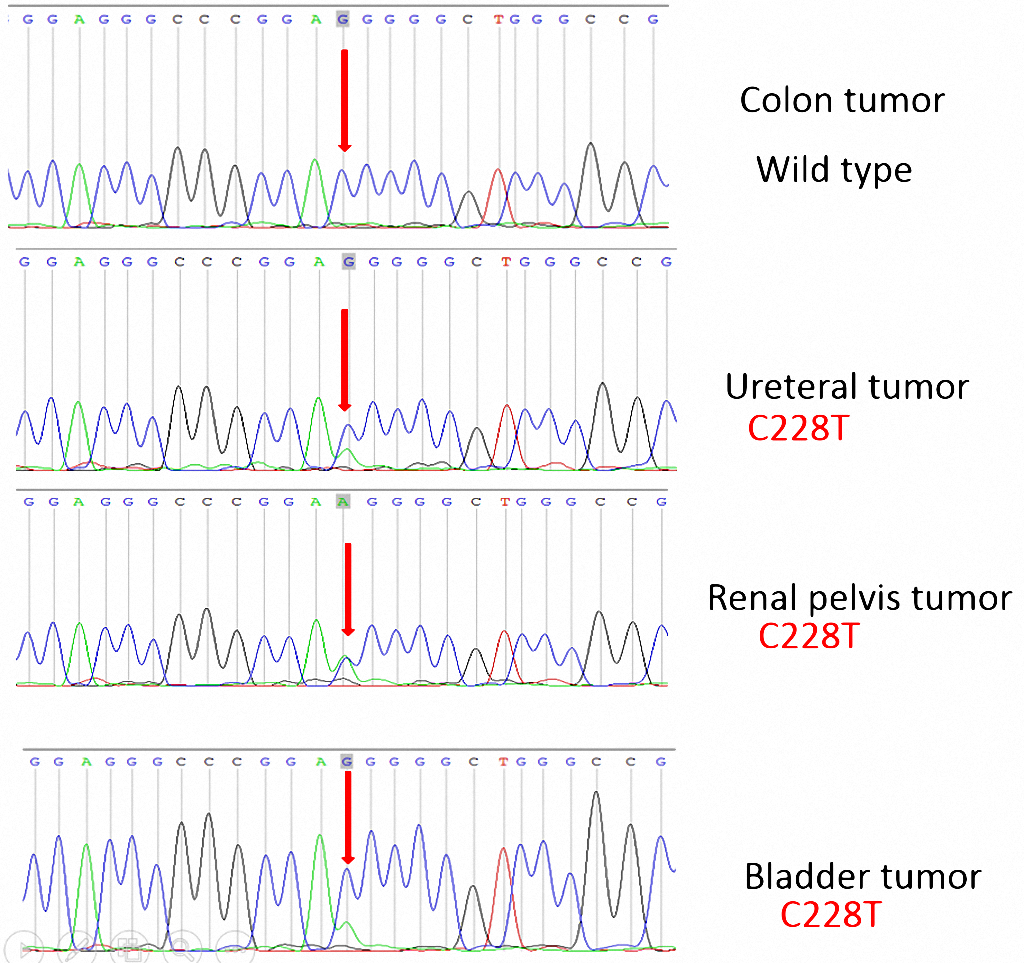

Supplement: Additional file 2: Figure S2. — Electropherogram of the telomerase reverse transcriptase (TERT) promoter region. (TIF 3855 kb) [file 13256_2016_879_MOESM2_ESM.tif]

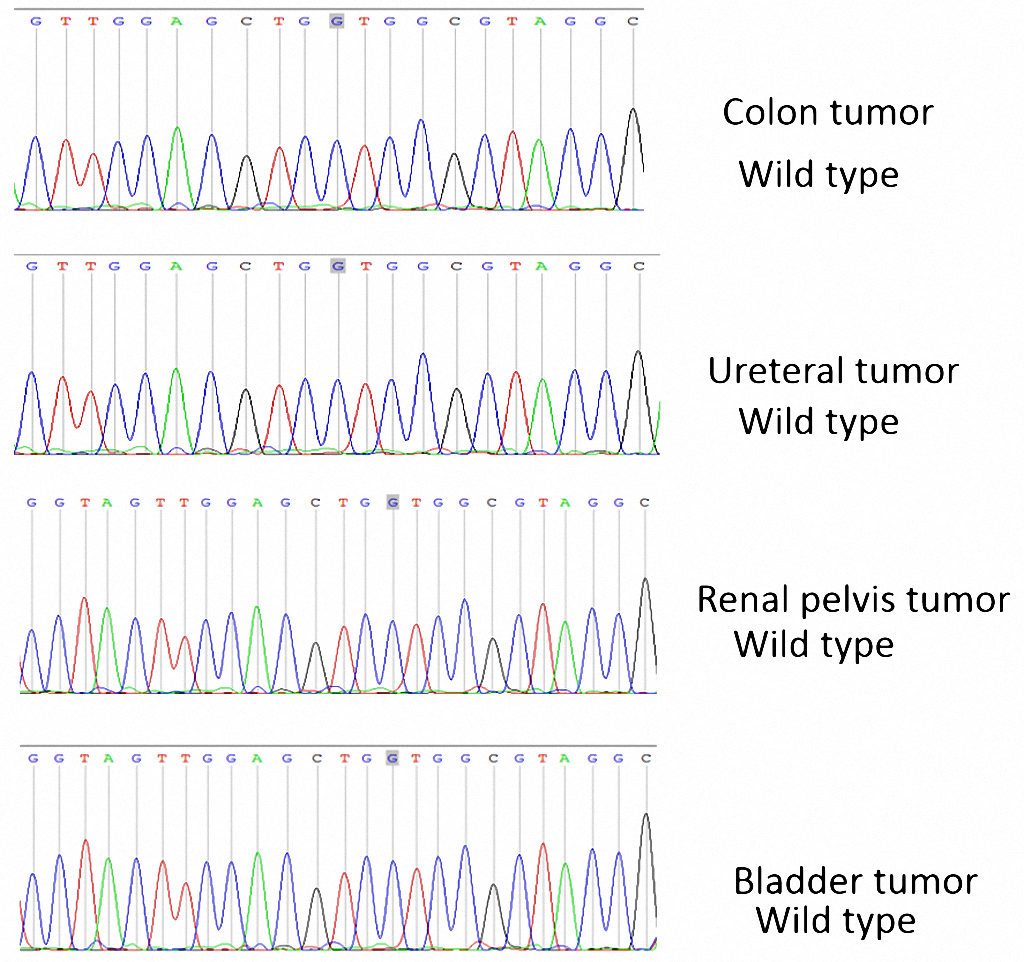

Supplement: Additional file 3: Figure S3. — Electropherogram of exon 2 of the KRAS gene. (TIF 3851 kb) [file 13256_2016_879_MOESM3_ESM.tif]
