# Supplementary material for: Epidermal growth factor receptor inhibitor with fluorouracil, leucovorin, and irinotecan as an alternative treatment for advanced upper tract urothelial carcinoma: a case report
Source: J Med Case Rep. 2016 Apr 18;10:98. doi: 10.1186/s13256-016-0879-6 (PMC4835853; doi:10.1186/s13256-016-0879-6)
Supplement: Additional file 4: — Supporting information. (DOCX 16 kb) [file 13256_2016_879_MOESM4_ESM.docx]

**Supporting Information**

Genomic DNA of the tumor tissues was extracted after standard proteinase K digestion with a Qiagen kit (QIAamp FFPE Tissue Kit). Tissue sections containing more than 20% tumor cells in the tissue block were selected according to the corresponding H&E stained sections. The telomerase reverse transcriptase (TERT) promoter region and exon 2 of the *KRAS* gene were amplified with polymerase chain reaction (primers for TERT promoter: CAGCGCTGCCTGAAACTC and GTCCTGCCCCTTCACCTT; primers for *KRAS* exon 2: GTGTGACATGTTCTAATATAGTCA and AGAATGGTCCTGCACCAGAATTAT; Go Taq G2 DNA polymerase, Promega). The quality of PCR products was confirmed with gel electrophoresis. Direct sequencing of PCR products was performed using a Big Dye terminator v3.1 cycle sequencing ready reaction kit (Applied Biosystems). Mutations were identified through bidirectional Big Dye sequencing.
